# Large-scale screening identifies enzyme combinations that remove *in situ* grown oral biofilm

**DOI:** 10.1016/j.bioflm.2024.100229

**Published:** 2024-10-04

**Authors:** Signe Maria Nielsen, Karina Kambourakis Johnsen, Lea Benedicte Skov Hansen, Pernille Dukanovic Rikvold, Andreas Møllebjerg, Lorena Gonzalez Palmén, Thomas Durhuus, Sebastian Schlafer, Rikke Louise Meyer

**Affiliations:** a Interdisciplinary Nanoscience Center (iNANO), Faculty of Natural Sciences, Aarhus University, Gustav Wieds Vej 14, 8000, Aarhus C, Denmark; b Section for Oral Ecology, Cariology, Department of Dentistry and Oral Health, Aarhus University, Vennelyst Boulevard 9, 8000, Aarhus C, Denmark; c Novonesis A/S, Biologiens Vej 2, 2800, Kgs. Lyngby, Denmark; d Department of Biology, Faculty of Natural Sciences, Aarhus University, Ny Munkegade 114, 8000, Aarhus C, Denmark

**Keywords:** Dental biofilm, Enzyme treatment, Mutanase, Dextranase, Glucanase, Confocal microscopy

## Abstract

Bacteria in the oral cavity are responsible for the development of dental diseases such as caries and periodontitis, but it is becoming increasingly clear that the oral microbiome also benefits human health. Many oral care products on the market are antimicrobial, killing a large part of the oral microbiome but without removing the disease-causing biofilm. Instead, non-biocidal matrix-degrading enzymes may be used to selectively remove biofilm without harming the overall microbiome.

The challenge of using enzymes to degrade biofilms is to match the narrow specificity of enzymes with the large structural diversity of extracellular polymeric substances that hold the biofilm together. In this study, we therefore perform a large-scale screening of single and multi-enzyme formulations to identify combinations of enzymes that most effectively remove dental biofilm.

We tested >400 different treatment modalities using 44 different enzymes in combinations with up to six enzymes in each formulation, on *in vitro* biofilms inoculated with human saliva. Mutanase was the only enzyme capable of removing biofilm on its own. Multi-enzyme formulations removed up to 69 % of the biofilm volume, and the most effective formulations all contained mutanase. We shortlisted 10 enzyme formulations to investigate their efficacy against biofilms formed on glass slabs on dental splints worn by 9 different test subjects. Three of the ten formulations removed more than 50 % of the biofilm volume. If optimal enzyme concentration and exposure time can be reached *in vivo*, these enzyme combinations have potential to be used in novel non-biocidal oral care products for dental biofilm control.

## 1 Introduction

The oral microbiome plays an important role in both health and disease [[1], [2], [3], [4]]. On the one hand, metabolic activity of oral bacteria produces metabolites that lower the blood pressure and prevent metabolic disorders [5,6]. On the other hand, some oral bacteria cause oral diseases like caries, gingivitis, periodontitis, and dysbiosis in the oral microbiome is linked to development of systemic diseases, such as diabetes and atherosclerosis [[7], [8], [9], [10]]. Microorganisms colonize non-shedding tooth surfaces where they assemble into biofilms also known as dental plaque. The biofilm on clinically healthy teeth is dominated by moderately acidogenic species such as non-mutans streptococci and *Actinomyces* spp., which do not cause disease. However, unhindered biofilm growth and frequent exposure to dietary carbohydrates results in a shift in the bacterial community towards oral dysbiosis with an increased prevalence of highly acidogenic and aciduric bacteria that contribute to caries development [11].

Current strategies for preventing oral biofilm diseases beyond mechanical cleaning and fluoride rely on antimicrobials, such as chlorhexidine, cetylpyridinium chloride, or metal ions, which are added to many oral care products. These compounds are mainly effective against planktonic bacteria and have limited effect on established biofilms [12]. The unspecific killing of microorganisms by antimicrobial products disrupts microbial homeostasis in the mouth and leads to a lowered bacterial diversity, which opens the space for pathogens [13,14]. Novel oral care products should therefore aim at disrupting pathogenic biofilms rather than killing the commensal microbiota. Non-biocidal matrix-degrading enzymes represent a promising therapeutic approach to control and limit the buildup of dental biofilm, as they disrupt the extracellular matrix that surrounds the microorganisms and thereby destabilize the biofilm whilst leaving commensals unscathed [15,16].

The biofilm matrix consists of a complex mixture of macromolecules, including polysaccharides, proteins, lipids and extracellular DNA [17]. These extracellular polymeric substances (EPS) stabilize the biofilm mechanically and mediate surface adhesion and cell-to-cell interactions. The biofilm matrix also creates chemical and physical microenvironments, such as areas with acidic pH, which are crucial for the development of caries [18,19]. Previous studies have successfully employed single enzymes, or a combination of 2–3 enzymes to remove laboratory-grown biofilms [16,[20], [21], [22]]. However, laboratory biofilm models are much less diverse than biofilms in the oral cavity, and results from such laboratory models probably translate poorly to *in vivo* application [23,24]. Given the complexity of the dental biofilm matrix and the multitude of interactions between different matrix components, it is unlikely that a single enzyme will be effective. Most notably, the structure and composition of extracellular polysaccharides varies from species to species and even from strain to strain within the same species [25], and polysaccharide-degrading enzymes are highly specific towards distinct glycosidic bonds. For example, mutanase cleaves α-1,3 D-glucosidic bonds which are highly present in the biofilm matrix produced by oral streptococci, while amylase cleaves α-1,4 D-glucosidic bonds, and dextranase cleaves α-1,6 D-glucosidic bonds. Polysaccharides can also interact closely with extracellular DNA [[26], [27], [28]], and proteins also contribute to matrix stability [29]. Identification of matrix-degrading enzyme solutions may therefore profit from a broad screening approach to evaluate a large array of enzyme combinations.

Here, we investigated the effect of two commercial multi-enzyme formulations and 44 single enzymes tested alone or in formulations with up to six enzymes that target important constituents of the biofilm matrix. We first quantified their biofilm-removing capability using a multispecies *in vitro* biofilm model inoculated with human saliva. With >400 treatment modalities, this is the largest screening of enzyme formulations against oral biofilms to date. We identified 10 lead formulations and evaluated these on biofilms grown *in situ* in the oral cavity, revealing 3 effective enzyme combinations that remove oral biofilms by degrading extracellular matrix components.

## 2 Methods

### 2.1 Enzymes and experimental strategy

All enzymes were dissolved in 50 mM HEPES with 100 mM NaCl pH 7 and stored at either −20 °C (mutanase) or 4 °C (all other enzymes). Enzyme stock solutions (150 mg/L) were prepared in artificial tap water (ATW: 2.1 mM CaCl_2_, 1.5 mM MgCl_2_ and 4 mM NaHCO_3_ in autoclaved milliQ water, sterile filtered) and administered in concentrations ranging from 5 to 75 mg/L. These concentrations reflect the concentration of purified enzymes.

A total of 44 different enzymes and two commercial formulations were selected based on their likelihood to target components of the extracellular matrix of oral biofilm and subsequently tested for their ability to remove *in vitro* biofilms grown from human saliva inoculum in combinations of 1–6 enzymes together (Table S1). From the initial 44 enzymes, 8 enzymes (Table 1) were selected and tested systematically in 85 different combinations at a fixed concentration of 15 mg/L. Ten enzyme formulations consisting of three to six different enzymes were then selected for evaluation of their ability to remove biofilms grown *in situ* in the oral cavity.

**Table 1:** Enzymes selected for evaluation further investigation after initial evaluation of 87 different enzyme formulations.

Enzyme	EC number	Suggested target	Chemical bond
Dextranase	EC 3.2.1.11	Dextran	α-1,6 D-glucosidic
Amylase	EC 3.2.1.1	Starch	α-1,4 D-glucosidic
Glucanase	EC 3.2.1.6	Glucan	α-1,3 - α-1,4 - α-1,6 D-glucosidic
Cellulase	EC 3.2.1.4	Cellulose	β-1,4 D-glucosidic
Xylanase	EC 3.2.1.8	Xylan	β-1,4 xylan
DNase	EC 3.1.21	DNA	Phosphodiester (DNA)
Mutanase	EC 3.2.1.59	Mutan	α-1,3 D-glucosidic
Lipase	EC 3.1.1	Lipids	triglycerides

All enzymes were produced by Novonesis A/S (Lyngby, Denmark). Some of the enzymes are experimental enzymes that are not commercially available and can only be obtained from Novonesis A/S under a confidentiality agreement.

### 2.2 *In vitro* biofilm growth and enzyme treatment

#### 2.2.1 Saliva inoculum

Pooled stimulated saliva from 10 healthy participants was used for inoculation of *in vitro*-grown biofilms, and for supplementing the laboratory media for biofilm growth. Saliva was collected and processed as described previously [30]. In brief, paraffin-stimulated saliva was collected on ice, pooled, cleared and either mixed with glycerol and phosphate buffered saline (PBS) (saliva:glycerol:PBS = 1:1:1) and stored at −80 °C, or else sterile filtered as described [31] and stored at −20 °C.

#### 2.2.2 In vitro biofilm growth from saliva inoculum

*In vitro* biofilms were grown in round bottom 96-well plates (Nunclon Delta Surface, Thermo Fischer Scientific, Waltham, MA, USA). Wells for biofilm growth were filled with 160 μL brain heart infusion broth (BHI) supplemented with 4 % sucrose, 20 μL sterile saliva and 20 μL saliva inoculum. Plates were incubated at 37 °C with light shaking at 50 rpm for 6, 12 or 24 h before enzyme treatment. The media was not exchanged until enzyme treatment was performed. See Ref. [32] for characterization of the microbial composition of 24 h old biofilms.

#### 2.2.3 Enzyme treatment of in vitro-grown biofilm

**Fig. 1:**
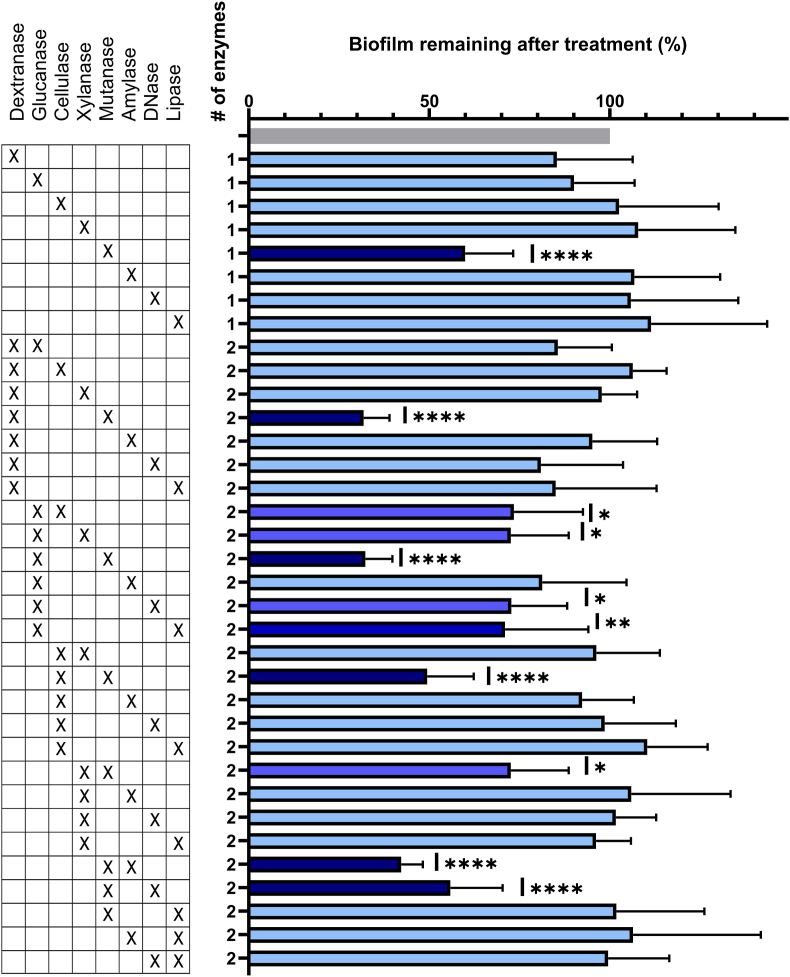
Single- and dual-enzyme treatment of *in vitro*-grown biofilm. Biofilms (24 h old, inoculated from human saliva) were treated for 2 h with 15 mg/L of each enzyme. The bar graph shows % biofilm remaining after the treatment, quantified by crystal violet staining, as compared to control treatment with artificial tap water. Darker shade of blue indicates increasing biofilm removal by the treatment. Error bars show standard deviations of the mean. The most effective single enzyme was mutanase, and the most effective dual-enzyme combination was mutanase combined with either dextranase or glucanase. Statistical significance was determined by one-way ANOVA followed by Dunnett's test. P-values below 0.05 were considered statistically significant. ∗: p = 0.0177–0.0386, ∗∗: p = 0.0011–0.0065, ∗∗∗: p = 0.0005, ∗∗∗∗: p < 0.0001. (For interpretation of the references to colour in this figure legend, the reader is referred to the Web version of this article.)

**Fig. 2:**
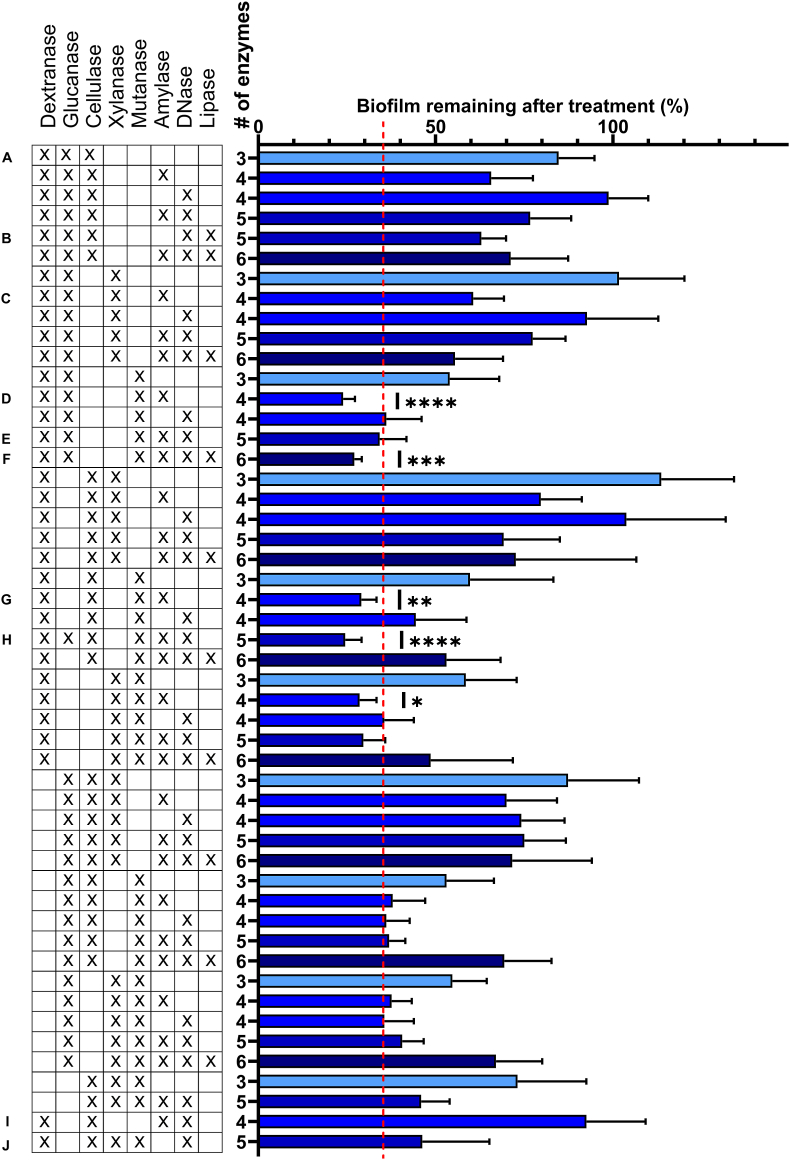
Multi-enzyme treatment of *in vitro*-grown biofilms. Biofilms (24 h old, inoculated from human saliva) were treated for 2 h with 15 mg/L of each enzyme. The bar graph shows % biofilm remaining after the treatment, quantified by crystal violet staining, as compared to control treatment with artificial tap water. Error bars show standard deviations of the mean. Darker shade of blue indicates increasing biofilm removal by the treatment. The results are benchmarked against the most effective dual-enzyme treatment (red line). Statistical significance between multiple-enzyme treatment and the best dual-enzyme combination was determined by one-way ANOVA followed by Dunnett's test. Multi-enzyme treatment did not increase biofilm removal. (For interpretation of the references to colour in this figure legend, the reader is referred to the Web version of this article.)

**Fig. 3:**
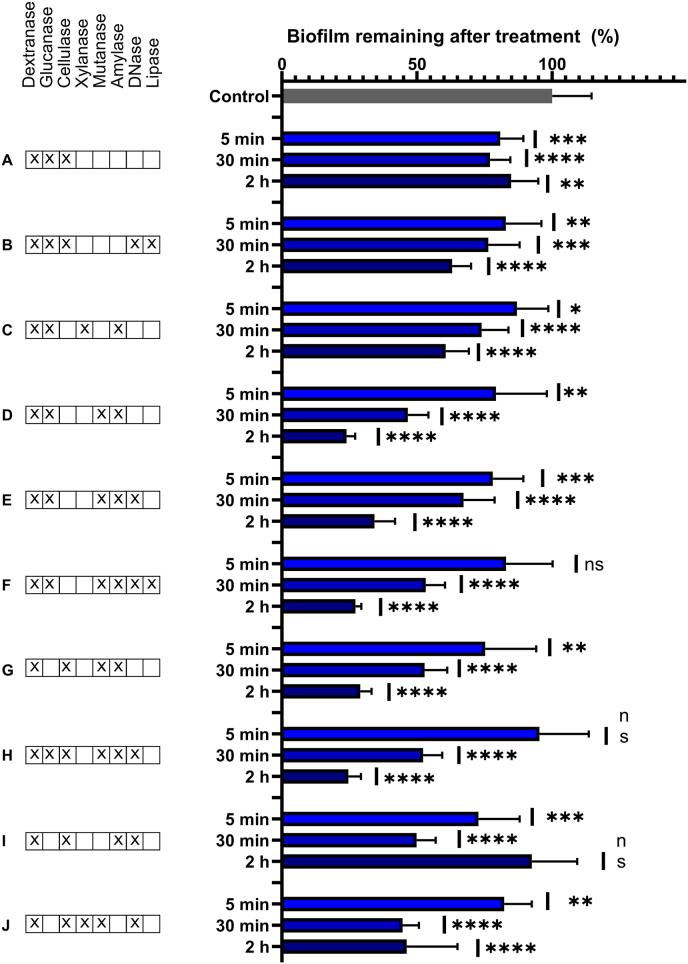
Effect of treatment duration for ten selected enzyme combinations on *in vitro-*grown biofilms. Biofilms (24 h old, inoculated from human saliva) were treated for 2 h, 30 min or 5 min with 15 mg/L of each enzyme. The bar graph shows % biofilm remaining after the treatment, quantified by crystal violet staining, compared to control treatment with artificial tap water. Error bars show standard deviations of the mean. Eight out of ten formulations reduced the amount of *in vitro* biofilm after 5 min of treatment, and most formulations were more effective after longer treatment. Statistical significance was determined by one-way ANOVA followed by Dunnett's test. P-values below 0.05 were considered statistically significant. ∗: p = 0.0156–0.0238, ∗∗: p = 0.0016–0.007, ∗∗∗: p = 0.0002–0.0007, ∗∗∗∗: p < 0.0001. (For interpretation of the references to colour in this figure legend, the reader is referred to the Web version of this article.)

After biofilm growth, the remaining liquid was gently removed by pipetting without disturbing the biofilm. The biofilm was treated with single enzymes and combinations of up to seven enzymes (Supplementary Table S1), in concentrations of 5 μg/mL – 75 μg/mL per enzyme. Enzymes were added by transferring 20 μL of stock solution (150 μg/mL) of each enzyme to the wells, followed by addition of ATW to a final volume of 200 μL. Untreated controls received ATW only. Enzyme treatment was carried out at 37 °C and with gentle shaking at 50 rpm. Standard treatment times were 2 h (Fig. 1, Fig. 2, Fig. 3), but shorter treatments (30 min and 5 min) were also tested (Fig. 3). After enzyme treatment, liquid was removed from the wells by pipetting, and the wells were rinsed three times with milliQ water. Enzyme treatment was performed in three biological replicates with four technical replicates each.

#### 2.2.4 Biofilm quantification of *in vitro* grown biofilm

After treatment, the remaining biofilm biovolume was quantified by CV staining. The biofilm was washed three times in 200 μL ATW, and the plate was shaken (slow setting, 17 Hz, 1020 rpm) for 5 s in a plate reader (BioTek PowerWave X52, Holm & Halby, Brøndby, Denmark), to remove unbound biofilm. Water was removed from the wells by inverting the plate over a funnel or paper towel. The plate was dried at 37 °C for 30 min, and the biofilm was then stained with 200 μL CV solution (Gram's crystal violet solution, Sigma-Aldrich, Burlington, USA) for 10 min at room temperature (RT). Unbound CV was rinsed off using demineralized water and the plates were emptied and dried at 37 °C for 30 min. After drying, the remaining CV bound to the biofilm was extracted in 200 μl acetic acid (33 %) for 30 min at RT and transferred to a flat-bottom 96-well microtiter plate. Absorbance was measured at 590 nm.

### 2.3 *In situ-*grown biofilm collection and treatment with multiple-enzyme formulations

Ten lead enzyme formulations from *in vitro* testing were selected for *ex vivo* treatment of *in situ-*grown biofilms (Fig. 2, formulations A-J). Nine healthy volunteers with no clinical signs of active caries or periodontitis were enrolled in the study. The absence of caries and periodontal disease was assessed using the Nyvad criteria [33] and the periodontal screening index [34]. *In situ* biofilms were grown on custom-made non-fluorescent glass slabs (4 x 4 × 1.5 mm, Menzel, Braunschweig, Germany), mounted on lower jaw splints specifically designed for each participant, as described previously [32]. The participants wore the splints for periods of 48 h, and only removed them during the intake of food or liquids other than water, and during dental hygiene procedures. During the growth periods, participants dipped the splint in 10 % (w/V) sucrose solution for 2 min, three times a day. After 48 h, the glass slabs with the *in situ-*grown biofilm were removed from the splint and taken directly to the laboratory for enzyme treatment. All ten enzyme formulations were tested on *in situ*-grown biofilms from each participant. The participants wore the splints for five experimental periods of 48 h, and from each of the five periods, nine glass slabs were collected from each participant for enzyme treatment. Each run provided biofilms for testing two enzyme combinations (three glass slabs per enzyme combination) and a corresponding control (three glass slabs). The biofilms were enzyme treated (see below) for 2 h and the biofilm biovolumes quantified by confocal microscopy. The study protocol was reviewed and approved by the Central Jutland Regional Committee on Health Research Ethics (1-10-72-285-18) and registered in the internal database of research projects at Aarhus University.

#### 2.3.1 Enzyme treatment of in situ-grown biofilm

Glass slabs with biofilms were randomly assigned to either enzyme or control treatment, washed three times in PBS and placed in round-bottom 96-well plates containing the selected enzyme formulations (Fig. 3, formulations A-J, 15 mg/L) or ATW (controls). Biofilms were enzyme treated for 2 h at 37 °C with gentle shaking at 50 rpm, washed three times in PBS, fixed in 4 % PFA for 2 h at RT, washed again three times in PBS, and stored in 1:1 PBS:ethanol at −20 °C until microscopy analysis.

#### 2.3.2 Quantification of in situ-grown biofilm

Prior to staining, glass slabs were rinsed in milliQ water and dried at RT. Exopolysaccharides in the biofilm were stained with 20 μL of ConcanavalinA (ConA) labeled with fluorescein isothiocyanate (FITC; 100 μM, Sigma-Aldrich) for 30 min in the dark, rinsed with PBS and dried at RT. Bacteria were stained with 30 μM propidium iodide (PI; Thermo Fischer Scientific, Waltham, MA, USA) for 15 min. After staining, glass slabs were placed with the biofilms facing down in 96-well plates for microscopy (Ibidi, GmbH, Martinsried, Germany) and imaged by confocal laser scanning microscopy (CLSM) using a 63x oil immersion objective (NA = 1.4; alpha Plan-Apochromat, Carl Zeiss, Germany). PI was excited at 555 nm and detected from 584 to 800 nm; ConA-FITC was excited at 488 nm and detected from 300 to 584 nm. The pinhole was set to 1 Airy Unit. For each biofilm, z-stacks (3 slices) spanning the height of the biofilms were acquired in 6 predefined fields of view (FOV).

Image files obtained by CLSM (.czi files) were converted to.tif and exported into the software daime [35]. Images were segmented by intensity thresholding, determining the area covered with bacteria. Microbial biovolumes were calculated according to the Cavalieri principle [36] by multiplying the total area of all three images in a stack with the interslice distance.

### 2.4 Statistical analysis

**Fig. 4:**
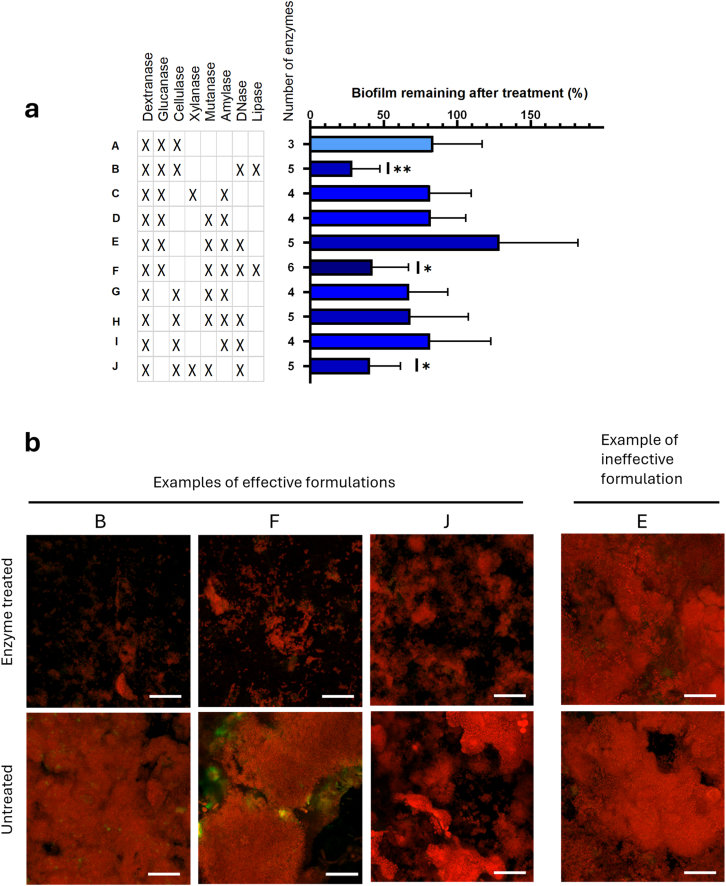
**Enzyme treatment of *in situ*-grown biofilms.** Biofilms were grown for 48 h on glass slabs in the oral cavity with three daily exposures to sucrose, and then treated *in vitro* with enzyme formulations (15 mg/L of each enzyme) for 2 h. The amount of biofilm was quantified by 3D imaging and biovolume calculation by digital image analysis. **a)**: Bar graph shows biofilm remaining (%) after enzyme treatment. Error bars = standard deviations of the mean. Biofilm removal was obtained by formulations B (p = 0.0059), F (p = 0.0388) and J (p = 0.0309). Statistical significance was determined by one-way ANOVA followed by Dunnett's test. P-values below 0.05 were considered statistically significant. **b)**: Representative confocal microscopy images of enzyme-treated biofilms and untreated controls. Bacteria were stained with propidium iodide (red) and extracellular polysaccharides were labeled with fluorescently conjugated Concanavalin A (green) which binds to α-D-mannosyl and α-D-glucosyl groups in the EPS. Scale bars = 20 μm. (For interpretation of the references to colour in this figure legend, the reader is referred to the Web version of this article.)

The effect of enzyme treatment on removal of *in vitro*-grown biofilm was quantified by CV staining (Fig. 1, Fig. 4; Table S1). For each treatment group, mean absorbance was calculated compared to control biofilms treated with ATW. Normal distribution of the data was verified by Shapiro Wilk tests and the effect of enzyme treatment was analyzed by one-way ANOVA with Dunnett's correction for multiplicity. One-way ANOVA with Dunnett's correction was also used to benchmark the effect of multi-enzyme formulations on *in vitro*-grown biofilm against the best dual-enzyme formulation (Fig. 2). For *in situ-*grown biofilms, the mean bacterial biovolume, determined by CLSM, was calculated for each subject and treatment group. Normal distribution and homogeneity of variance were assessed as described above, and one-way ANOVA with Dunnett's correction was used to determine the effect of enzyme treatment on biofilm removal. Statistical analyses were performed using Prism (GraphPad Prism version 9.0.0 for Windows, GraphPad Software, San Diego, California USA, www.graphpad.com). P-values below 0.05 were considered statistically significant.

## 3 Results and discussion

### 3.1 Eight enzymes partially remove *in vitro* biofilms

We first evaluate if enzymes removed *in vitro-*grown biofilms inoculated from human saliva and grown in BHI supplemented with sucrose and sterile saliva. Initially, 321 enzyme treatments were performed using different combinations of 44 enzymes, different enzyme concentrations (5–75 mg/L) and biofilms grown for either 6 or 12 h (Table S1). Enzymes removed up to 79 % of the biofilm biomass as estimated by crystal violet staining. We selected eight enzymes for further evaluation: Glucanase, dextranase, mutanase, cellulase, xylanase, amylase, DNase, and lipase (Table 1). These enzymes were re-evaluated as single enzymes and in systematic combinations of up to 6 enzymes at a fixed concentration (15 mg/L) for 2 h against 24 h-old *in vitro-*grown biofilms (Fig. 1, Fig. 2).

#### 3.1.1 Treatments with one or two enzymes point to mutanase, dextranase and glucanase as most effective

Only mutanase removed a statistically significant part (40 %) of the biofilm (Fig. 1). Some enzymes appeared ineffective on their own but did remove biofilms in combination with other enzymes. For example, glucanase had no effect on its own but removed up to 69 % of the biofilm when combined with cellulase, xylanase, DNase or mutanase. Except for the combination of mutanase and lipase, all dual-enzyme formulations containing mutanase removed a statistically significant part of the biofilm. Mutanase/dextranase and mutanase/glucanase combinations were the most effective, removing up to 69 % of the biofilm. Enzyme formulations that lacked mutanase, dextranase and glucanase had no effect (Fig. 1).

#### 3.1.2 Treatment with 3 or more enzymes in combination did not increase the effect on in vitro-grown biofilm

We then combined multiple enzymes to target a wider range of components in the biofilm's extracellular matrix, using the same experimental procedure as before (Fig. 2). Despite targeting a broader range of matrix components, treatments with 3–6 enzymes did not increase biofilm removal compared to the mutanase-dextranase combination (when correcting for type I errors). Mutans and dextrans are considered the most important dental plaque matrix polysaccharides [37], and it is therefore not surprising that the best-performing enzymes were those that hydrolyse α-1,3 glucosidic bonds (mutanase) and α-1,6 glucosidic bonds (dextranase). Several other studies have also demonstrated the effect of mutanase and/or dextranase on mono- or multispecies streptococcal biofilms grown in laboratory media with 1 % sucrose [21,38,39], grown with intermittent sucrose exposures [40], or biofilms enriched from human saliva in media containing sucrose [41]. Mutans are produced by streptococci, and these are often selected for in *in vitro* biofilm models [42] where aerobic growth conditions and high nutrient- and sucrose levels are optimal for biofilm formation by fast-growing, aerobic streptococci [43]. Although we used a diverse inoculum (human saliva), our growth conditions enriched for streptococci, which dominated the biofilm after 24 h [32]. Streptococci use sucrose as a precursor for production of extracellular mutans and dextrans, and it is therefore not surprising that the 4 % sucrose in our growth media leads to biofilms rich in streptococci and mutans. More complex biofilms may benefit from combination of several carbohydrases to tackle a mixture of different types of polysaccharides or different degrees of branching, which can occur even among the mutans produced by streptococci [44].

#### 3.1.3 Selection of enzymes for further investigations

We selected 10 enzyme formulations for further investigation using *in situ-*grown dental biofilms collected from healthy participants. Keeping in mind the bias of our *in vitro* biofilm model, we selected enzyme formulations based on their biofilm-removing properties as well as formulations that contained different classes of enzymes. The selected formulations are noted A-J in Fig. 2. Four formulations were selected based on their effect on *in vitro*-grown biofilms (formulations D, E, G and H in Fig. 2) while six formulations were included to ensure a broad variety of enzyme combinations (formulations A, B, C, F, I and J in Fig. 2). Dental biofilms harbor a diverse bacterial community, and the biofilm extracellular matrix does not only consist of biopolymers synthesized by the bacteria, but also components from dietary sources and the host. We therefore included lipases and DNases despite their poor effect on *in vitro-*grown biofilms.

#### 3.1.4 The effect of enzyme treatment is time-dependent

To impact oral health, enzymes must remain in the oral cavity for long enough to degrade the biofilm. We did not measure if enzymes remain active after rinsing the biofilms, but we proceeded to test if the formulations removed biofilms at shorter treatment times of 30 and 5 min. This investigation was performed with the ten selected enzyme formulations on *in vitro-*grown biofilms prepared as in previous experiments.

Even at shorter treatment times, most enzyme formulations reduced the amount of *in vitro*-grown biofilm, although the effect was less pronounced (Fig. 3). Formulation I (dextranase, cellulase, amylase and DNase) was the most effective formulation at 5 min of treatment with 28 % biofilm reduction. At 30 min treatment, formulation J (dextranase, cellulase, xylanase, mutanase and DNase) was the most effective with 52 % biofilm removal, which was similar to the result from 2 h of treatment.

The retention time of matrix-degrading enzymes in the oral cavity is unknown. Studies of antimicrobial mouth wash products show a residual retention of 25–30 % after each 1-min treatment, and they remain active for up to 6 h [45]. Chlorhexidine has a retention time of up to 11 h, with a drop-off at 6 h. Here, the dental pellicle and oral mucosa were the main reservoirs [[46], [47], [48]]. It is therefore promising that all 10 tested enzyme formulations affected *in vitro*-grown biofilms after 30 min of treatment. Despite this encouraging result, we chose to perform 2 h of enzyme treatment in experiments involving *in situ-*grown biofilms where the large biological variation may obscure effects of the enzyme treatment.

### 3.2 Treatment of *in situ*-grown biofilms shows therapeutic potential

To test the therapeutic potential of multiple-enzyme treatment on *in vivo-*grown biofilms, nine healthy volunteers wore individually designed lower-jaw splints equipped with glass carriers for biofilm collection. The splints were worn for 48 h during which the biofilms were exposed to sucrose 3 times per day. Glass carriers were then removed from the splints and treated for 2 h with enzyme formulations A-J, before biofilm quantification by CLSM. Treatment with enzyme formulations B, F and J showed a promising therapeutic potential as they all reduced the volume of the biofilm by more than 50 % (Fig. 4a + b).

Interestingly, the enzyme formulations with greatest efficacy against *in vitro-*grown biofilms were not always those that performed best on *in situ*-grown biofilms. In fact, only one of the three formulations that removed *in situ*-grown biofilms had shown a similar effect *in vitro*. The best-performing formulation was formulation B (dextranase, glucanase, cellulase, DNase and lipase), which reduced the biofilm volume by 71 % (p = 0.0062) (Fig. 4a + b). This formulation did not contain mutanase and had only a moderate effect on *in vitro*-grown biofilms (28 % reduction; Fig. 2). Formulations F (dextranase, glucanase, mutanase, amylase, DNase and lipase), and J (dextranase, cellulase, xylanase, mutanase and DNase) removed 58 % (p = 0.0062) and 60 % (p = 0.003) of the biofilms, respectively. Interestingly, two out of three effective formulations contained lipase, which points to the importance of dietary and host lipids for dental biofilm stability, a subject that merits further investigation. Dextranase and DNase were common denominators in all three formulations. This is in line with other studies that identify eDNA and dextrans as key components of the biofilm matrix in oral biofilms [49,50].

The observed differences between treatment of *in vitro-* and *in situ*-grown biofilms underline that *in vitro* biofilms are simplified models of oral biofilms with a different species and matrix composition. While the *in vitro* biofilm model is dominated by *streptococci* (>95 %) [32], the *in situ-*grown biofilm model is more diverse with *Streptococcus* spp comprising approx. 25–75 % and other genera, such as *Haemophilus, Gemella, Fusobacterium, Veillonella, Prevotella, Granulicatella, Rothia* and *Neisseria* present in varying amounts [32]. Given the higher complexity of the *in situ*-grown biofilm, it is not surprising that its removal requires a greater variety of matrix-degrading enzymes, while a single enzyme, such as mutanase, can disrupt the *Streptococcus-*dominated *in vitro-*grown biofilm [51]. The biofilm composition varies from person to person as shown in a previous characterization of the *in situ* biofilm model [32]. Hence, one of the great challenges of targeting the EPS matrix of oral biofilms is that the composition and spatial organization of the biofilm matrix depends on the bacterial species composition [25]. Current knowledge of the extracellular matrix composition of oral biofilms is limited [52,53]. A better understanding of the polymicrobial EPS matrix could provide crucial knowledge of why – and why not – specific enzymes and enzyme combinations are effective at removing biofilm *in vivo.* As reflected in our results, a one-fits-all enzyme formulation to remove oral biofilms *in vivo* must contain a cocktail of diverse enzymes to accommodate the large variation in biofilm composition and structure among different people.

### 3.3 Non-biocidal treatment with multiple enzymes is a promising therapeutic approach for biofilm removal

While many oral care products focus on antimicrobial properties [54], our findings show that a non-biocidal approach with matrix-degrading enzymes has considerable potential as a new therapeutic avenue for oral biofilm control. The present study provides proof-of-concept that treatment with enzyme formulations that contain a cocktail of different enzymes can remove not only *in vitro*-grown biofilms, but also biofilms grown in the oral cavity. Only one previous study has tested enzyme solutions against *in situ* grown oral biofilms, and demonstrated that a mixture of proteases could remove such biofilm [55]. However, proteolytic activity is not desirable in the oral cavity due to the risk of damage to the mucosal surfaces, and this risk is probably the reason why such proteases have not been pursued for this application since the study was published in 2001.

It is a limitation to our study that we can only disclose the general enzyme classification and not the specific mechanism and species of origin for each enzyme, since these are experimental enzymes that are not yet commercially available, and the further details are subject to confidentiality. Nevertheless, the efficacy of each enzyme class provides an overview of the types of biopolymers that are important to oral biofilms, and the study shows a potential for applying enzymes in oral care products.

The future development of such commercial products for dental biofilm control could supplement standard oral care by combatting biofilms in between tooth brushing or in places that are hard to reach for mechanical cleaning. Development of such products will depend on several other factors that have not been investigated in the present study, such as the activity and shelf-life of the enzymes in oral care product formulations, the biocompatibility of enzyme formulations, and the effect of enzyme treatment on disease-related biofilms from caries-active patients or periodontitis patients. Although being non-biocidal, the effect of enzyme treatment on the commensal oral microbiota remains to be explored.

In conclusion, this study is the first to investigate the effect of enzyme treatment of oral biofilms using a broad selection of matrix-degrading enzymes a systematic manner. This approach enables direct comparison of different enzyme classes and different formulations that combine multiple enzymes. Using a pool of 44 enzymes, we tested the effect of >400 different treatments on the removal of *in vitro*-grown biofilm and identified 10 lead formulations of 3–6 enzymes for removal of *in vivo*-grown biofilms. Three of these 10 formulations removed 60–70 % of the biomass of biofilms grown *in situ* in the oral cavity and thus show a therapeutic potential for dental biofilm control. How this treatment affects the microbial composition and cariogenic properties of biofilms will be the focus of future research.

## CRediT authorship contribution statement

**Signe Maria Nielsen:** Writing – original draft, Visualization, Methodology, Investigation, Formal analysis, Data curation. **Karina Kambourakis Johnsen:** Writing – review & editing, Investigation, Formal analysis. **Lea Benedicte Skov Hansen:** Writing – review & editing, Investigation, Formal analysis. **Pernille Dukanovic Rikvold:** Writing – review & editing, Investigation. **Andreas Møllebjerg:** Writing – review & editing, Investigation, Formal analysis. **Lorena Gonzalez Palmén:** Writing – review & editing, Resources, Project administration, Funding acquisition, Conceptualization. **Thomas Durhuus:** Writing – review & editing, Resources, Project administration, Funding acquisition, Conceptualization. **Sebastian Schlafer:** Writing – review & editing, Supervision, Resources, Project administration, Methodology, Investigation, Funding acquisition, Formal analysis, Data curation, Conceptualization. **Rikke Louise Meyer:** Writing – review & editing, Writing – original draft, Supervision, Resources, Project administration, Investigation, Formal analysis, Conceptualization, Writing – review & editing, Supervision, Resources, Methodology, Investigation, Funding acquisition, Conceptualization.

## Declaration of competing interest

The authors declare the following financial interests/personal relationships which may be considered as potential competing interests:

Signe Maria Nielsen reports financial support and equipment, drugs, or supplies were provided by Novonesis AS. Pernille T Rikvold reports financial support was provided by Novonesis AS. Andreas Mollebjerg reports financial support was provided by Novonesis AS. Lea Benedicte Skov Hansen reports a relationship with Novonesis AS that includes: employment. Lorena Gonzalez Palmen reports a relationship with Novonesis AS that includes: employment. Thomas Durhuus reports a relationship with Novonesis AS that includes: employment. Corresponding author is an editor of the journal. If there are other authors, they declare that they have no known competing financial interests or personal relationships that could have appeared to influence the work reported in this paper.

## Data Availability

Data will be made available on request.
