# Supplementary material for: Large-scale screening identifies enzyme combinations that remove in situ grown oral biofilm
Source: Biofilm. 2024 Oct 4;8:100229. doi: 10.1016/j.bioflm.2024.100229 (PMC11740801; doi:10.1016/j.bioflm.2024.100229)
Supplement: Multimedia component 1 [file mmc1.docx]

**Table S1.** Screening of enzymes to identify single- and multi-enzyme formulations capable of removing in vitro-grown biofilms enriched from human saliva. Different enzyme concentrations were tested against 6 h and 12 h old biofilms in ≥ 3 replicates. Values show means and standard devliation. Heat map indicates most effective enzymes in green and least effective in red. The results were used to select 8 enzymes for further analysis (indicated with *).

| **Enzyme (* indicated selected for further analysis)** | **Target** | **Sample ID** | **Concentration (per enzyme in multi-enzyme formulations) (mg/L)** | **Biofilm age (h)** | **Treatment duration (h)** | **Biofilm remaining after treatment (%)** | **standard deviation (%)** |
| --- | --- | --- | --- | --- | --- | --- | --- |
| Protease/Amylase/Pectate lyase formulation 1 | multiple components matrix | 1 | 75 | 6 | 2 | 89 | 5 |
|  |  |  | 25 | 6 | 2 | 89 | 9 |
| Protease/Amylase/Pectate lyase formulation 2 | multiple components matrix | 2 | 75 | 6 | 2 | 77 | 10 |
|  |  |  | 25 | 6 | 2 | 77 | 6 |
| Dextranase* | dextran/EPS | 3 | 75 | 6 | 2 | 64 | 9 |
|  |  |  | 25 | 6 | 2 | 60 | 13 |
|  |  |  | 75 | 12 | 2 | 101 | 23 |
|  |  |  | 25 | 12 | 2 | 81 | 19 |
|  |  |  | 75 | 12 | 2 | 71 | 14 |
|  |  |  | 25 | 12 | 2 | 83 | 10 |
| Mannanase | mannan/EPS | 4 | 75 | 6 | 2 | 93 | 6 |
|  |  |  | 25 | 6 | 2 | 98 | 22 |
|  |  |  | 75 | 12 | 2 | 115 | 13 |
|  |  |  | 25 | 12 | 2 | 106 | 3 |
| Pectate lyase | pectin/EPS | 5 | 75 | 6 | 2 | 133 | 18 |
|  |  |  | 25 | 6 | 2 | 119 | 5 |
| Amylase* | starch/matrix | 6 | 75 | 6 | 2 | 123 | 14 |
|  |  |  | 25 | 6 | 2 | 98 | 23 |
|  |  |  | 75 | 12 | 2 | 144 | 16 |
|  |  |  | 25 | 12 | 2 | 106 | 29 |
|  |  |  | 75 | 12 | 2 | 82 | 8 |
|  |  |  | 25 | 12 | 2 | 106 | 2 |
| Glucanase* | glucans/EPS | 7 | 75 | 6 | 2 | 73 | 10 |
|  |  |  | 25 | 6 | 2 | 66 | 10 |
|  |  |  | 75 | 12 | 2 | 72 | 13 |
|  |  |  | 25 | 12 | 2 | 107 | 25 |
|  |  |  | 75 | 12 | 2 | 87 | 5 |
|  |  |  | 25 | 12 | 2 | 79 | 7 |
| Muramidase | N-acetylmuramide glycanhydrolase | 8 | 75 | 6 | 2 | 75 | 7 |
|  |  |  | 25 | 6 | 2 | 86 | 6 |
| Lipase | lipids | 9 | 75 | 6 | 2 | 102 | 19 |
|  |  |  | 25 | 6 | 2 | 112 | 12 |
| Cellulase* | cellulose/EPS | 10 | 75 | 6 | 2 | 77 | 11 |
|  |  |  | 25 | 6 | 2 | 90 | 6 |
|  |  |  | 75 | 12 | 2 | 130 | 8 |
|  |  |  | 25 | 12 | 2 | 76 | 17 |
|  |  |  | 75 | 12 | 2 | 87 | 4 |
|  |  |  | 25 | 12 | 2 | 95 | 12 |
| Xylanase* | xylans/EPS | 11 | 75 | 6 | 2 | 115 | 18 |
|  |  |  | 25 | 6 | 2 | 88 | 19 |
|  |  |  | 75 | 12 | 2 | 97 | 13 |
|  |  |  | 25 | 12 | 2 | 80 | 20 |
|  |  |  | 75 | 12 | 2 | 81 | 4 |
|  |  |  | 25 | 12 | 2 | 90 | 14 |
| DNAse* | DNA | 12 | 75 | 6 | 2 | 115 | 11 |
|  |  |  | 25 | 6 | 2 | 99 | 16 |
|  |  |  | 75 | 12 | 2 | 66 | 9 |
|  |  |  | 25 | 12 | 2 | 89 | 13 |
|  |  |  | 75 | 12 | 2 | 125 | 13 |
|  |  |  | 25 | 12 | 2 | 144 | 29 |
| Dispersin* | PNAG/EPS | 13 | 75 | 6 | 2 | 111 | 19 |
|  |  |  | 25 | 6 | 2 | 119 | 14 |
| DNAse | DNA | 14 | 75 | 6 | 2 | 133 | 10 |
|  |  |  | 25 | 6 | 2 | 114 | 12 |
|  |  |  | 75 | 12 | 2 | 87 | 5 |
|  |  |  | 25 | 12 | 2 | 87 | 3 |
| DNAse | DNA | 15 | 75 | 6 | 2 | 80 | 14 |
|  |  |  | 25 | 6 | 2 | 91 | 6 |
|  |  |  | 75 | 12 | 2 | 92 | 4 |
|  |  |  | 25 | 12 | 2 | 85 | 4 |
| DNAse | DNA | 16 | 75 | 6 | 2 | 100 | 13 |
|  |  |  | 25 | 6 | 2 | 56 | 10 |
|  |  |  | 75 | 12 | 2 | 95 | 7 |
|  |  |  | 25 | 12 | 2 | 97 | 7 |
| Mutanase* | mutan/EPS | 17 | 75 | 6 | 2 | 21 | 9 |
|  |  |  | 25 | 6 | 2 | 19 | 4 |
|  |  |  | 25 | 12 | 2 | 72 | 5 |
|  |  |  | 10 | 12 | 2 | 43 | 6 |
|  |  |  | 75 | 12 | 2 | 24 | 4 |
|  |  |  | 25 | 12 | 2 | 18 | 2 |
|  |  |  | 75 | 12 | 2 | 56 | 9 |
|  |  |  | 25 | 12 | 2 | 64 | 14 |
|  |  |  | 75 | 12 | 2 | 40 | 6 |
|  |  |  | 25 | 12 | 2 | 50 | 13 |
|  |  |  | 10 | 12 | 2 | 48 | 23 |
|  |  |  | 15 | 12 | 2 | 42 | 17 |
|  |  |  | 5 | 12 | 2 | 37 | 5 |
|  |  |  | 15 | 12 | 2 | 41 | 8 |
|  |  |  | 5 | 12 | 2 | 60 | 5 |
| Mannanase | mannan/EPS | 19 | 75 | 6 | 2 | 80 | 11 |
|  |  |  | 25 | 6 | 2 | 104 | 17 |
| PelA | pel polysaccharide | 20 | 75 | 6 | 2 | 101 | 22 |
|  |  |  | 25 | 6 | 2 | 112 | 18 |
| DNAse | DNA | 21 | 75 | 6 | 2 | 114 | 8 |
|  |  |  | 25 | 6 | 2 | 100 | 32 |
|  |  |  | 75 | 12 | 2 | 89 | 4 |
|  |  |  | 25 | 12 | 2 | 98 | 9 |
| DNAse | DNA | 22 | 75 | 6 | 2 | 129 | 14 |
|  |  |  | 25 | 6 | 2 | 150 | 8 |
|  |  |  | 75 | 12 | 2 | 60 | 18 |
|  |  |  | 25 | 12 | 2 | 66 | 14 |
|  |  |  | 75 | 12 | 2 | 94 | 10 |
|  |  |  | 25 | 12 | 2 | 108 | 19 |
| DNAse | DNA | 23 | 75 | 6 | 2 | 126 | 8 |
|  |  |  | 25 | 6 | 2 | 113 | 17 |
|  |  |  | 75 | 12 | 2 | 82 | 8 |
|  |  |  | 25 | 12 | 2 | 82 | 2 |
| DNAse | DNA | 24 | 75 | 6 | 2 | 122 | 8 |
|  |  |  | 25 | 6 | 2 | 103 | 20 |
|  |  |  | 75 | 12 | 2 | 85 | 2 |
|  |  |  | 25 | 12 | 2 | 90 | 2 |
| DNAse | DNA | 25 | 75 | 6 | 2 | 99 | 19 |
|  |  |  | 25 | 6 | 2 | 77 | 5 |
|  |  |  | 75 | 12 | 2 | 88 | 3 |
|  |  |  | 25 | 12 | 2 | 88 | 2 |
| Mannanase | mannan/EPS | 26 | 15 | 12 | 2 | 108 | 6 |
|  |  |  | 5 | 12 | 2 | 92 | 10 |
| Mannanase | mannan/EPS | 27 | 15 | 12 | 2 | 84 | 17 |
|  |  |  | 5 | 12 | 2 | 90 | 25 |
| Lipase* | lipids | 28 | 15 | 12 | 2 | 63 | 17 |
|  |  |  | 5 | 12 | 2 | 76 | 24 |
| Cellulase* | cellulose/EPS | 29 | 15 | 12 | 2 | 52 | 5 |
|  |  |  | 5 | 12 | 2 | 104 | 8 |
| Xylanase | xylans/EPS | 31 | 15 | 12 | 2 | 87 | 16 |
|  |  |  | 5 | 12 | 2 | 87 | 4 |
| Amylase | starch/matrix | 32 | 15 | 12 | 2 | 100 | 9 |
|  |  |  | 5 | 12 | 2 | 105 | 4 |
| Phospholipase | phospholipids | 33 | 15 | 12 | 2 | 104 | 4 |
|  |  |  | 5 | 12 | 2 | 97 | 8 |
| Phospholipase | phospholipids | 34 | 15 | 12 | 2 | 111 | 4 |
|  |  |  | 5 | 12 | 2 | 86 | 24 |
| Phospholipase | phospholipids | 35 | 15 | 12 | 2 | 104 | 6 |
|  |  |  | 5 | 12 | 2 | 101 | 4 |
| Phospholipase | phospholipids | 36 | 15 | 12 | 2 | 96 | 29 |
|  |  |  | 5 | 12 | 2 | 104 | 5 |
| RNAse | RNA | 37 | 15 | 12 | 2 | 98 | 14 |
|  |  |  | 5 | 12 | 2 | 110 | 8 |
| Mannanase | mannan/EPS | 38 | 15 | 12 | 2 | 100 | 14 |
|  |  |  | 5 | 12 | 2 | 77 | 15 |
| PelA | pel polysaccharide | 39 | 15 | 12 | 2 | 87 | 19 |
|  |  |  | 5 | 12 | 2 | 104 | 10 |
| Xylanase | xylans/EPS | 40 | 15 | 12 | 2 | 94 | 19 |
|  |  |  | 5 | 12 | 2 | 83 | 18 |
| Cutinase | cutin | 41 | 15 | 12 | 2 | 101 | 8 |
|  |  |  | 5 | 12 | 2 | 95 | 12 |
| Cellulase | cellulose/EPS | 42 | 15 | 12 | 2 | 90 | 9 |
|  |  |  | 5 | 12 | 2 | 86 | 6 |
| Mutanase | mutan/EPS | 43 | 15 | 12 | 2 | 77 | 7 |
|  |  |  | 5 | 12 | 2 | 89 | 8 |
| Mutanase | mutan/EPS | 44 | 15 | 12 | 2 | 77 | 6 |
|  |  |  | 5 | 12 | 2 | 81 | 3 |
| Rhamnogalacturonidase | Rhamno-galacturan | 45 | 15 | 12 | 2 | 93 | 12 |
|  |  |  | 5 | 12 | 2 | 87 | 1 |
| Galactosaminogalactanase | Galactosamin-Galactan | 46 | 15 | 12 | 2 | 71 | 9 |
|  |  |  | 5 | 12 | 2 | 79 | 4 |
| Dextranase, Mannase |  | 3+4 | 75+75 | 12 | 2 | 93 | 21 |
|  |  |  | 25+25 | 12 | 2 | 99 | 20 |
| Dextranase, Amylase |  | 3+6 | 75+75 | 12 | 2 | 106 | 32 |
|  |  |  | 25+25 | 12 | 2 | 80 | 36 |
|  |  |  | 75+75 | 12 | 2 | 101 | 24 |
|  |  |  | 25+25 | 12 | 2 | 101 | 16 |
| Dextranase, Glucanase |  | 3+7 | 75+75 | 12 | 2 | 80 | 24 |
|  |  |  | 25+25 | 12 | 2 | 50 | 17 |
|  |  |  | 75+75 | 12 | 2 | 50 | 12 |
|  |  |  | 25+25 | 12 | 2 | 47 | 7 |
|  |  |  | 75+75 | 12 | 2 | 57 | 22 |
|  |  |  | 25+25 | 12 | 2 | 56 | 19 |
|  |  |  | 75+75 | 12 | 2 | 27 | 7 |
|  |  |  | 25+25 | 12 | 2 | 20 | 5 |
|  |  |  | 75+75 | 12 | 2 | 43 | 4 |
|  |  |  | 75+25 | 12 | 2 | 63 | 14 |
|  |  |  | 25+75 | 12 | 2 | 42 | 4 |
|  |  |  | 25+25 | 12 | 2 | 38 | 7 |
|  |  |  | 75+75 | 12 | 2 | 55 | 7 |
|  |  |  | 25+25 | 12 | 2 | 76 | 4 |
|  |  |  | 15+15 | 12 | 2 | 42 | 17 |
|  |  |  | 5+5 | 12 | 2 | 37 | 5 |
| Dextranase, Cellulase |  | 3+10 | 75+75 | 12 | 2 | 65 | 1 |
|  |  |  | 25+25 | 12 | 2 | 70 | 4 |
|  |  |  | 75+75 | 12 | 2 | 45 | 13 |
|  |  |  | 25+25 | 12 | 2 | 63 | 13 |
|  |  |  | 75+75 | 12 | 2 | 47 | 6 |
|  |  |  | 25+25 | 12 | 2 | 49 | 11 |
|  |  |  | 75+75 | 12 | 2 | 53 | 7 |
|  |  |  | 75+25 | 12 | 2 | 74 | 4 |
|  |  |  | 25+75 | 12 | 2 | 49 | 12 |
|  |  |  | 25+25 | 12 | 2 | 79 | 3 |
|  |  |  | 15+15 | 12 | 2 | 84 | 6 |
|  |  |  | 5+5 | 12 | 2 | 68 | 22 |
| Dextranase, Xylanase |  | 3+11 | 75+75 | 12 | 2 | 81 | 4 |
|  |  |  | 25+25 | 12 | 2 | 82 | 6 |
|  |  |  | 75+75 | 12 | 2 | 40 | 5 |
|  |  |  | 25+25 | 12 | 2 | 42 | 15 |
|  |  |  | 75+75 | 12 | 2 | 63 | 8 |
|  |  |  | 75+25 | 12 | 2 | 79 | 10 |
|  |  |  | 25+75 | 12 | 2 | 61 | 16 |
|  |  |  | 25+25 | 12 | 2 | 82 | 14 |
|  |  |  | 15+15 | 12 | 2 | 77 | 16 |
|  |  |  | 5+5 | 12 | 2 | 75 | 19 |
| Dextranase, Cellulase |  | 3+29 | 15+15 | 12 | 2 | 67 | 13 |
|  |  |  | 5+5 | 12 | 2 | 56 | 3 |
| Mannanase, Amylase |  | 4+6 | 75+75 | 12 | 2 | 83 | 3 |
|  |  |  | 25+25 | 12 | 2 | 101 | 3 |
| Mannanase, Glucanase |  | 4+7 | 75+75 | 12 | 2 | 77 | 9 |
|  |  |  | 25+25 | 12 | 2 | 80 | 6 |
| Mannanase, Cellulase |  | 4+10 | 75+75 | 12 | 2 | 76 | 4 |
|  |  |  | 25+25 | 12 | 2 | 93 | 3 |
| Mannanase, Xylanase |  | 4+11 | 75+75 | 12 | 2 | 97 | 3 |
|  |  |  | 25+25 | 12 | 2 | 85 | 7 |
| Amylase, Glucanase |  | 6+7 | 75+75 | 12 | 2 | 66 | 4 |
|  |  |  | 25+25 | 12 | 2 | 92 | 10 |
|  |  |  | 75+75 | 12 | 2 | 52 | 10 |
|  |  |  | 25+25 | 12 | 2 | 47 | 18 |
|  |  |  | 75+75 | 12 | 2 | 121 | 24 |
|  |  |  | 25+25 | 12 | 2 | 132 | 42 |
|  |  |  | 75+75 | 12 | 2 | 41 | 16 |
|  |  |  | 25+25 | 12 | 2 | 43 | 4 |
|  |  |  | 15+15 | 12 | 2 | 57 | 17 |
|  |  |  | 5+5 | 12 | 2 | 89 | 4 |
| Amylase, Cellulase |  | 6+10 | 75+75 | 12 | 2 | 87 | 7 |
|  |  |  | 25+25 | 12 | 2 | 98 | 8 |
| Amylase, Xylanase |  | 6+11 | 75+75 | 12 | 2 | 160 | 66 |
|  |  |  | 25+25 | 12 | 2 | 101 | 4 |
| Glucanase, Cellulase |  | 7+10 | 75+75 | 12 | 2 | 86 | 19 |
|  |  |  | 25+25 | 12 | 2 | 81 | 4 |
|  |  |  | 75+75 | 12 | 2 | 88 | 21 |
|  |  |  | 25+25 | 12 | 2 | 60 | 9 |
|  |  |  | 75+75 | 12 | 2 | 45 | 4 |
|  |  |  | 75+25 | 12 | 2 | 64 | 14 |
|  |  |  | 25+75 | 12 | 2 | 44 | 4 |
|  |  |  | 25+25 | 12 | 2 | 40 | 7 |
|  |  |  | 15+15 | 12 | 2 | 84 | 26 |
|  |  |  | 5+5 | 12 | 2 | 111 | 19 |
| Glucanase, Xylanase |  | 7+11 | 75+75 | 12 | 2 | 61 | 12 |
|  |  |  | 25+25 | 12 | 2 | 55 | 18 |
|  |  |  | 75+75 | 12 | 2 | 66 | 10 |
|  |  |  | 25+25 | 12 | 2 | 51 | 14 |
|  |  |  | 75+75 | 12 | 2 | 86 | 26 |
|  |  |  | 25+25 | 12 | 2 | 100 | 29 |
|  |  |  | 15+15 | 12 | 2 | 87 | 15 |
|  |  |  | 5+5 | 12 | 2 | 96 | 17 |
| Glucanase, Cellulase |  | 7+29 | 15+15 | 12 | 2 | 66 | 2 |
|  |  |  | 5+5 | 12 | 2 | 80 | 5 |
| Dextranase, Amylase, Glucanase |  | 3+6+7 | 75+75 | 12 | 2 | 78 | 26 |
|  |  |  | 25+25 | 12 | 2 | 100 | 13 |
|  |  |  | 75+75 | 12 | 2 | 77 | 13 |
|  |  |  | 25+25 | 12 | 2 | 67 | 13 |
| Dextranase, Xylanase, Cellulase |  | 3+7+10 | 75+75 | 12 | 2 | 71 | 29 |
|  |  |  | 25+25 | 12 | 2 | 75 | 18 |
|  |  |  | 75+75 | 12 | 2 | 54 | 13 |
|  |  |  | 25+25 | 12 | 2 | 67 | 6 |
| Dextranase, Glucanase, Xylanase |  | 3+7+11 | 75+75 | 12 | 2 | 64 | 20 |
|  |  |  | 25+25 | 12 | 2 | 83 | 20 |
|  |  |  | 75+75 | 12 | 2 | 62 | 11 |
|  |  |  | 25+25 | 12 | 2 | 60 | 17 |
| Dextranase, Glucanase, Mutanase |  | 3+7+17 | 75+75+75 | 12 | 2 | 12 | 1 |
|  |  |  | 75+75+25 | 12 | 2 | 11 | 2 |
|  |  |  | 75+75+10 | 12 | 2 | 13 | 2 |
|  |  |  | 15+15+15 | 12 | 2 | 14 | 4 |
|  |  |  | 5+5+5 | 12 | 2 | 10 | 2 |
| Dextranase, Glucanase, Dispersin |  | 3+7+13 | 25+25+25 | 12 | 2 | 63 | 6 |
|  |  |  | 25+25+10 | 12 | 2 | 58 | 9 |
| Dextranase, Glucanase, Mutanase |  | 3+7+43 | 15+15+15 | 12 | 2 | 48 | 6 |
|  |  |  | 5+5+5 | 12 | 2 | 42 | 4 |
| Dextranase, Glucanase, Mutanase |  | 3+7+44 | 15+15+15 | 12 | 2 | 44 | 9 |
|  |  |  | 5+5+5 | 12 | 2 | 55 | 7 |
| Dextranase, Cellulase, Mutanase |  | 3+10+17 | 15+15+15 | 12 | 2 | 16 | 1 |
|  |  |  | 5+5+5 | 12 | 2 | 25 | 4 |
| Dextranase, Cellulase, Mutanase |  | 3+10+43 | 15+15+15 | 12 | 2 | 30 | 11 |
|  |  |  | 5+5+5 | 12 | 2 | 33 | 10 |
| Dextranase, Cellulase, Mannanse |  | 3+10+4 | 15+15+15 | 12 | 2 | 47 | 16 |
|  |  |  | 5+5+5 | 12 | 2 | 42 | 13 |
| Dextranase, Xylanase, Mutanase |  | 3+11+17 | 15+15+15 | 12 | 2 | 21 | 4 |
|  |  |  | 5+5+5 | 12 | 2 | 20 | 7 |
| Dextranase, Mutanase, Cellulase |  | 3+17+29 | 15+15+15 | 12 | 2 | 61 | 3 |
|  |  |  | 5+5+5 | 12 | 2 | 68 | 7 |
| Dextranase, Cellulase, Mutanase |  | 3+29+43 | 15+15+15 | 12 | 2 | 39 | 11 |
|  |  |  | 5+5+5 | 12 | 2 | 39 | 5 |
| Dextranase, Cellulase, Mutanase |  | 3+29+44 | 15+15+15 | 12 | 2 | 58 | 4 |
|  |  |  | 5+5+5 | 12 | 2 | 67 | 2 |
| Amylase, Glucanase, Cellulase |  | 6+7+10 | 75+75+75 | 12 | 2 | 45 | 8 |
|  |  |  | 25+25+25 | 12 | 2 | 45 | 16 |
|  |  |  | 75+75+75 | 12 | 2 | 58 | 10 |
|  |  |  | 25+25+25 | 12 | 2 | 32 | 8 |
|  |  |  | 75+75+75 | 12 | 2 | 73 | 12 |
|  |  |  | 75+75+25 | 12 | 2 | 76 | 12 |
|  |  |  | 75+25+75 | 12 | 2 | 65 | 8 |
|  |  |  | 75+25+25 | 12 | 2 | 81 | 10 |
|  |  |  | 25+75+75 | 12 | 2 | 73 | 12 |
|  |  |  | 25+75+25 | 12 | 2 | 65 | 6 |
|  |  |  | 25+25+75 | 12 | 2 | 63 | 16 |
|  |  |  | 25+25+25 | 12 | 2 | 83 | 14 |
|  |  |  | 15+15+15 | 12 | 2 | 87 | 6 |
|  |  |  | 5+5+5 | 12 | 2 | 55 | 20 |
| Amylase, Glucanase, Xylanase |  | 6+7+11 | 75+75+75 | 12 | 2 | 63 | 19 |
|  |  |  | 25+25+25 | 12 | 2 | 92 | 25 |
| Amylase, Glucanase, Mutanase |  | 6+7+17 | 75+75+75 | 12 | 2 | 11 | 2 |
|  |  |  | 75+75+25 | 12 | 2 | 11 | 2 |
|  |  |  | 75+75+10 | 12 | 2 | 17 | 5 |
|  |  |  | 15+15+15 | 12 | 2 | 32 | 12 |
|  |  |  | 5+5+5 | 12 | 2 | 43 | 10 |
| Amylase, Glucanase, Cellulase |  | 6+7+29 | 15+15+15 | 12 | 2 | 65 | 9 |
|  |  |  | 5+5+5 | 12 | 2 | 62 | 3 |
| Amylase, Glucanase, Mutanase |  | 6+7+43 | 15+15+15 | 12 | 2 | 68 | 10 |
|  |  |  | 5+5+5 | 12 | 2 | 61 | 13 |
| Amylase, Glucanase, Mutanase |  | 6+7+44 | 15+15+15 | 12 | 2 | 31 | 15 |
|  |  |  | 5+5+5 | 12 | 2 | 47 | 16 |
| Glucanase, Cellulase, Mutanase |  | 7+10+17 | 15+15+15 | 12 | 2 | 24 | 5 |
|  |  |  | 5+5+5 | 12 | 2 | 35 | 2 |
| Glucanase, Mutanase, Cellulase |  | 7+17+29 | 15+15+15 | 12 | 2 | 44 | 4 |
|  |  |  | 5+5+5 | 12 | 2 | 61 | 6 |
| Mutanase, DNAse, DNase |  | 17+16+25 | 15+15+15 | 12 | 2 | 55 | 4 |
|  |  |  | 5+5+5 | 12 | 2 | 59 | 6 |
| Dextranase, Amylase, Glucanase, Cellulase |  | 3+6+7+10 | 75+75+75 | 12 | 2 | 81 | 7 |
|  |  |  | 25+25+25 | 12 | 2 | 63 | 15 |
| Dextranase, Glucanase, DNase, DNase |  | 3+7+16+25 | 25+25+25 | 12 | 2 | 50 | 12 |
|  |  |  | 25+25+1 | 12 | 2 | 80 | 3 |
| Dextranase, Amylase, Glucanase, Xylanase |  | 3+6+7+10+11 | 75+75+75 | 12 | 2 | 69 | 5 |
|  |  |  | 25+25+25 | 12 | 2 | 73 | 11 |
| Dextranase, Mannanase, Amylase, Glucanase, Xylanase |  | 3+4+6+7+10+11 | 75+75+75 | 12 | 2 | 50 | 9 |
|  |  |  | 25+25+25 | 12 | 2 | 72 | 12 |
| Dextranase, Mannanase, Amylase, Glucanase, Xylanase, DNase |  | 3+4+6+7+10+11+12 | 75+75+75 | 6 | 2 | 39 | 11 |
|  |  |  | 25+25+25 | 6 | 2 | 49 | 8 |
|  |  |  | 75+75+75 | 12 | 2 | 45 | 11 |
|  |  |  | 25+25+25 | 12 | 2 | 46 | 8 |
|  |  |  | 75+75+75 | 12 | 2 | 64 | 16 |
|  |  |  | 25+25+25 | 12 | 2 | 57 | 16 |
|  |  |  | 75+75+75 | 12 | 2 | 49 | 16 |
|  |  |  | 25+25+25 | 12 | 2 | 48 | 13 |
|  |  |  | 75+75+75 | 12 | 2 | 61 | 6 |
|  |  |  | 25+25+25 | 12 | 2 | 56 | 9 |
